# Real‐time dissolved carbon dioxide monitoring II: Surface aeration intensification for efficient CO_2_ removal in shake flasks and mini‐bioreactors leads to superior growth and recombinant protein yields

**DOI:** 10.1002/bit.27252

**Published:** 2020-01-09

**Authors:** Viki R. Chopda, Timothy Holzberg, Xudong Ge, Brandon Folio, Lynn Wong, Michael Tolosa, Yordan Kostov, Leah Tolosa, Govind Rao

**Affiliations:** ^1^ Department of Chemical, Biochemical and Environmental Engineering Center for Advanced Sensor Technology, University of Maryland Baltimore Maryland

**Keywords:** dissolved carbon dioxide, mini‐bioreactor, shake flasks, surface aeration intensification

## Abstract

Mass transfer is known to play a critical role in bioprocess performance and henceforth monitoring dissolved O_2_ (DO) and dissolved CO_2_ (dCO_2_) is of paramount importance. At bioreactor level these parameters can be monitored online and can be controlled by sparging air/oxygen or stirrer speed. However, traditional small‐scale systems such as shake flasks lack real time monitoring and also employ only surface aeration with additional diffusion limitations imposed by the culture plug. Here we present implementation of intensifying surface aeration by sparging air in the headspace of the reaction vessel and real‐time monitoring of DO and dCO_2_ in the bioprocesses to evaluate the impact of intensified surface aeration. We observed that sparging air in the headspace allowed us to keep dCO_2_ at low level, which significantly improved not only biomass growth but also protein yield. We expect that implementing such controlled smart shake flasks can minimize the process development gap which currently exists in shake flask level and bioreactor level results.

## Graphical abstract

Surface aeration intensification was implemented in shake flasks enabled with in house developed monitoring sensors for pH, dissolved oxygen and dissolved CO_2_. Efficient removal of CO_2_ from the shake flasks and enhanced O_2_ supply resulted in increased biomass growth and protein yield. Improved surface aeration in shake flasks can translate into better scale down models and smoother scale up activities, and thus will bridge the gap that exists in process development.

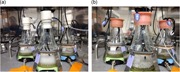

## 1 INTRODUCTION

The mass transfer including oxygen supply and dissolved CO_2_ (dCO_2_) stripping is one of the critical factors which affect the performance of aerobic bioprocesses across the scale from shake flask to manufacturing level (Matsunaga, Kano, Maki, & Dobashi, 2009). Although shake flask cultures are aerobic, O_2_ dissolved in the culture broth is generally insufficient for maximal cell growth. Similarly, high‐density cultures in the bioreactor suffer from poor dissolved oxygen (DO) concentration. To counter these issues, variations have been evaluated such as having inner concavities or convexities in the flask (baffled flask) to increase the oxygen diffusion (Büchs, 2001) and by supplying pure oxygen or increasing back pressure in the bioreactor (Priyanka, Roy, Chopda, Gomes, & Rathore, 2019). However, these variations have their own limitations such as the former one creates a shear to the cells and the later one generates oxidative stress to the cells, both of which negatively affect the cell growth and the product produced. Accumulation of dCO_2_ is another recurrent issue in large‐scale production bioreactors and mostly ignored in shake flask cultivation (Jenzsch, Gnoth, Kleinschmidt, Simutis, & Lübbert, 2007; Mostafa & Gu, 2003). Although a significant portion of dCO_2_ gets stripped through surface aeration in shake flasks, the rate of stripping is limited and largely determined by the closure material used and the liquid surface to volume ratio. Recently, a few case studies showed that dCO_2_ has a significant impact on small‐scale production systems (Takahashi & Aoyagi, 2018a, 2018b). However, due to the lack of reliable portable sensors for shake flask and mini‐bioreactor cultures, the parameters such as DO and dCO_2_ are rarely measured and their impact at this small scale is not clearly understood (Chopda, Gomes, & Rathore, 2016; Chopda, Pathak, Batra, Gomes, & Rathore, 2017; Gomes, Chopda, & Rathore, 2015). Due to the process analytical technology drive, the last decade has witnessed advances in shake flask monitoring sensors which led to improved understanding of the effect of various parameters on the culture growth and overall metabolism (Ge, Kostov, & Rao, 2005 &, 2006; Hanson et al., 2007; Kermis, Kostov, Harms, & Rao, 2002; Tolosa, Kostov, Harms, & Rao, 2002; Vallejos, Brorson, Moreira, & Rao, 2010). Various researchers proved that as the success of large‐scale operations is determined significantly by DO and dCO_2_ concentrations, similarly, these parameters have a significant role at small scale bioprocessing system too (Blombach & Takors, 2015). Conventionally, oxygen supply and dCO_2_ stripping are performed by gas sparging and agitation in large‐scale cultures, because of their large mass transfer rate and operational simplicity (Mitchell‐Logean & Murhammer, 1997). However, both gas sparging and agitation rates are restricted to low levels because of operational constraints (e.g., foaming, hydrodynamics) and biological limitations (e.g., shear sensitive cells). In case of shake flask, the culture is solely dependent on the orbital shaking and the limited surface aeration that occurs through the headspace for the DO supply and dCO_2_ stripping. In fact, in one such case study, Takahashi and Aoyagi (2018b) showed that intermittent opening of culture plugs temporarily changes the CO_2_ concentration in the headspace, which results in observable changes in microbial physiology. This indicates that in the culture vessel, the CO_2_ gets accumulated without proper ventilation and further forms a blanket (CO_2_ blanket theory), which hinders the effective gaseous mass transfer (Xing, Lewis, Borys, & Li, 2017). By intermittent opening, the culture plug may have allowed better gas transfer though temporarily, but it is significant enough to generate an observable impact on microbial physiology. This implicates that there is potential scope to optimize the shake flask and bioreactor cultures by regulating headspace gas distribution.

In this paper, we evaluated the impact of sparging air in the headspace of bio‐reaction vessels such as shake flasks and mini‐bioreactors using our novel rate‐based sensor for in‐situ dCO_2_ monitoring. By introducing air in the headspace, the gaseous distribution in the headspace and the dissolved gas concentration in the liquid broth are expected to change. Our investigation proved that the surface aeration plays a critical role in shake flask process development. With controlled surface aeration in the shake flask and mini‐bioreactor, we were able to improve not only the biomass growth but also the protein yield.

## 2 MATERIALS AND METHODS

### 2.1 *Yarrowia lipolytica* Po1g‐Leu fermentation

*Yarrowia lipolytica* is classified as an oleaginous yeast species because of its ability to accumulate lipids in large quantities (Xu, Qiao, Ahn, & Stephanopoulos, 2016). We used genetically modified versions of the Po1g strain engineered for flavonoid biosynthesis (Po1g with flavonoid pathway). The Po1g‐Leu strain was cultured in YPD broth. 300 µL of glycerol stock was added to 5 ml of YPD broth in 50 ml falcon tube and allowed to grow at 30°C and 250 rpm for 20‐24 hr. This preculture was further used to inoculate the mini‐bioreactor culture with the desired starting optical density. The volume of the mini‐bioreactor is 100 ml with a working volume of 50 ml. The bioreaction was conducted in batch mode at 30°C and lasted for 48 hr. To avoid excessive foaming, the mini‐bioreactor was bubble aerated at a flow rate of 20 cm^3^/min.

### 2.2 *Escherichia coli* fermentation

The gene *LivJ* from *E. coli* that codes for Leu/Ile/Val ABC transporter periplasmic binding protein was synthesized with a C‐terminus 6xHis tag. The synthesized fragment was inserted into the MCS of the expression vector pET28a between *Nco*I and *Xho*I. The A177C mutant was generated via site‐directed mutagenesis of the original construct. All plasmids were verified with sequencing. 300 µL of glycerol stock was added to 5 ml of Luria‐Bertani (LB) media broth in 50 ml falcon tube and allowed to grow at 37°C and 250 rpm for 20‐24 hr. This preculture was further used to inoculate the shake flask culture (50 ml culture in 250 ml flask) with the desired starting optical density at 200 rpm. At around 4 hr, 1 mM of IPTG was added as an inducer and at around 6 hr, a bolus of glucose feed (4 g/L culture) was added at once.

Cell pellets obtained from the fermentation process were thawed out on ice and resuspended with lysis buffer in the ratio 1 g of pellet to 10 ml of lysis buffer. The suspension was sonicated and then centrifuged at 10,000 rpm, 4°C to obtain the soluble fraction. Each sample of supernatant was added to a 10‐ml bed of Ni‐NTA resin that was pre‐equilibrated. After binding, the resin bed was subjected to the following: five column volumes of binding buffer, and eight column volumes of wash buffer. The protein of interest was then eluted in three fractions. Finally, the protein concentration was quantified using a standard Bradford assay (Kruger, 2009).

All the yeast and bacteria fermentation processes were monitored for pH and DO using optical sensors developed by our group for shake flasks and mini‐bioreactors (Scientific Industries Inc., Bohemia, NY). The details of the sensors can be found in the articles from our group (Hanson et al., 2007; Kermis et al., 2002; Kostov, Harms, Randers‐Eichhorn, & Rao, 2001; Tolosa et al., 2002). The dCO_2_ was monitored using the rate‐based sensor also developed by our group (Chatterjee et al., 2015; Chopda et al., 2019).

### 2.3 Surface aeration intensification

To intensify the surface aeration in the shake flask, standard‐size holes (ϕ3.15 mm) were made in the culture plug for air in and out. Additional ports were made for CO_2_ sensor in and out, and for sampling. The mini‐bioreactor has the necessary ports for air in and out, and for installing the needle‐shaped dCO_2_ probe. The flow rate of the air for overlay was set at 20 cm^3^/min and the delivery pressure was set at 10 psig from the source. Air was supplied from the source to the culture vessel using proprietary fluoroelastomer Versilon™ F‐5500‐A tubing of internal diameter 1.57 mm.

## 3 RESULTS

### 3.1 Impact of culture plug and overlay on shake flask fermentation

**Figure 1:**
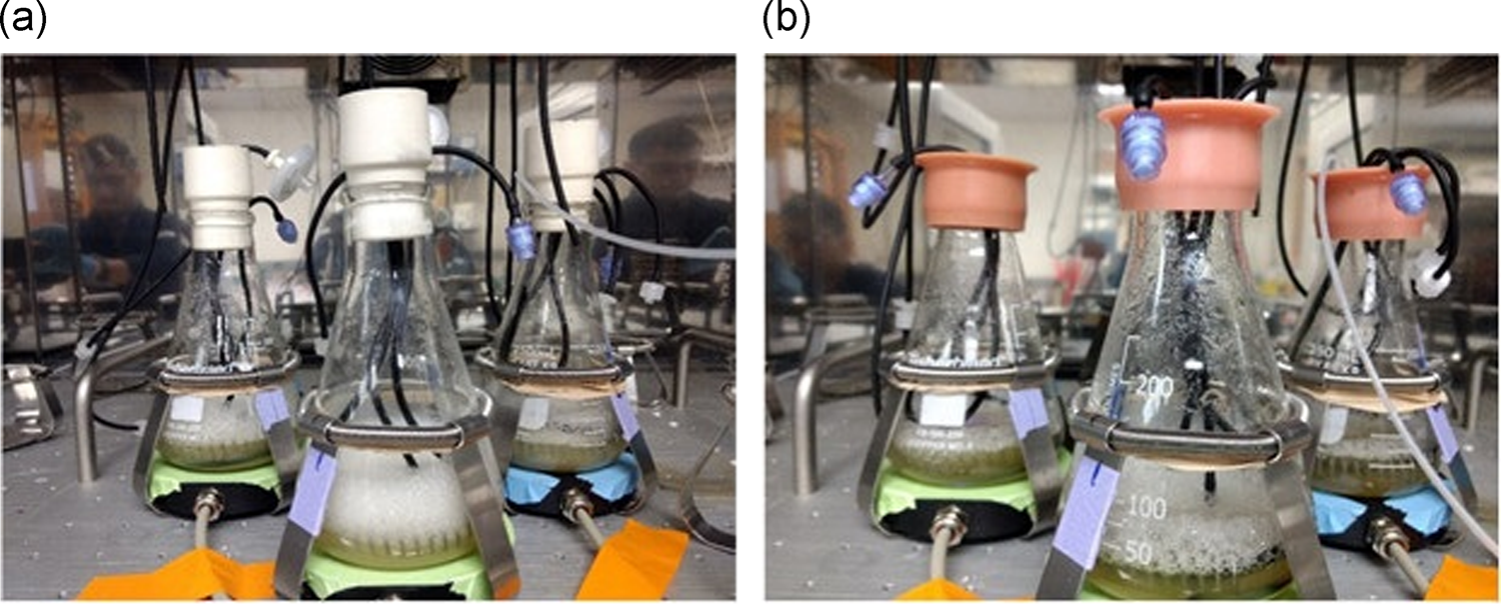
Demonstration of shake flask setup with coasters at the bottom for real‐time pH and dissolved oxygen (DO) monitoring: (a) Shake flask with rubber septum cap and (b) shake flask with sponge cap. The dissolved CO_2_ (dCO_2_) measurement loop was housed in the spring coil on the bottom [Color figure can be viewed at wileyonlinelibrary.com]

The culture plug used in shake flask has a significant role in gaseous exchange in the headspace. Traditionally, cotton plugs and sponge caps are commonly used for shake flasks (Amoabediny & Buchs, 2010; Takahashi & Aoyagi, 2018a, 2018b, 2018c). The culture plug type and with or without surface air intensification conditions are expected to have significant effects on the gaseous mass transfer by altering the gaseous distribution in the vessel. Here we used dCO_2_ as a monitoring parameter to evaluate the impact of culture plug and surface air intensification on the culture. Two different culture plugs, rubber septum cap and sponge cap, were evaluated using *E. coli* culture. The two experimental setups are demonstrated in Figure 1.

**Figure 2:**
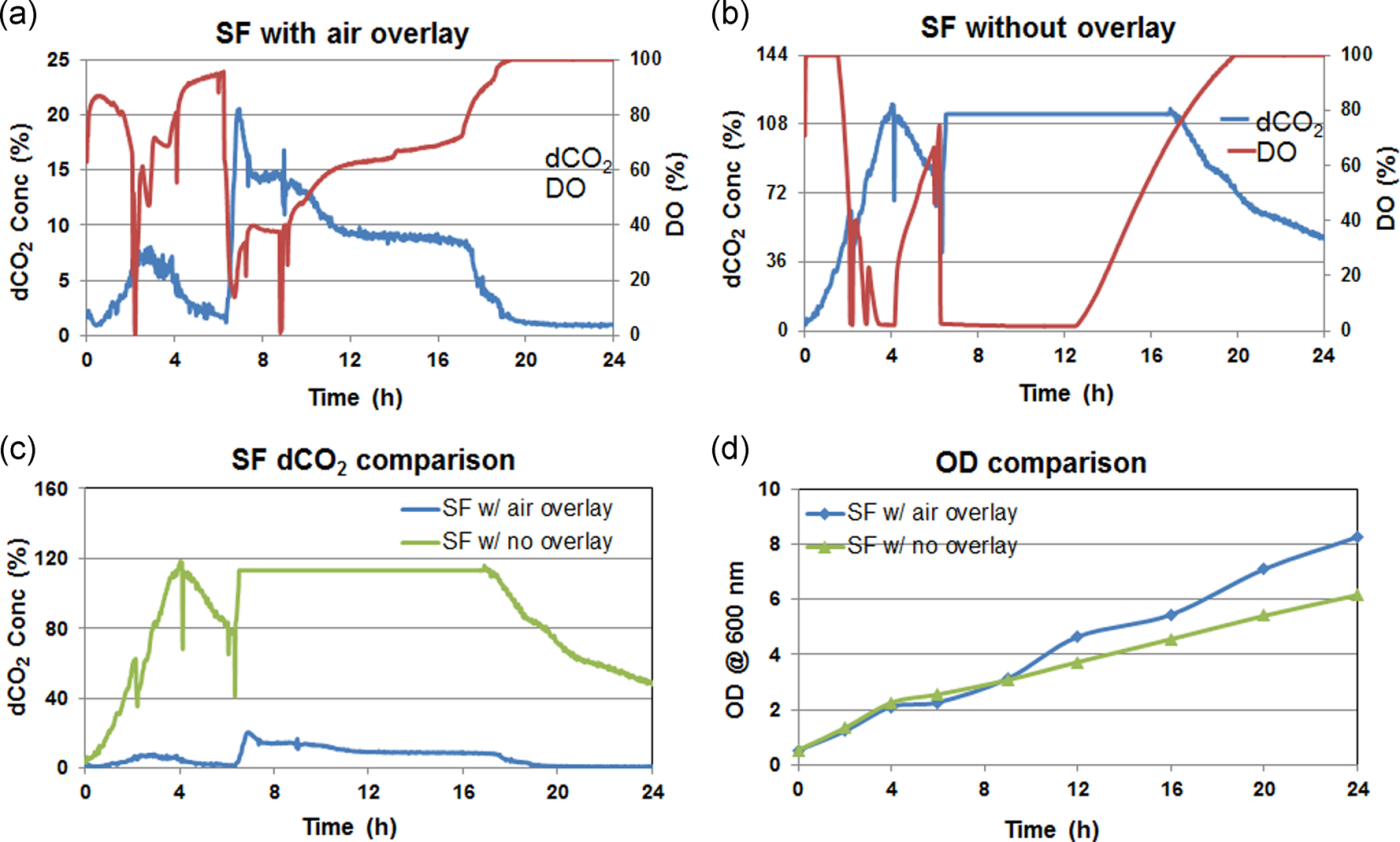
Process parameters monitored in *Escherichia coli* shake flask fermentation with rubber septum cap. (a) DO and dCO_2_ profiles with air overlay. (b) DO and dCO_2_ profiles with no overlay. (c) Comparison of dCO_2_ profiles under different overlay conditions showing the significance of surface aeration. (d) Comparison of biomass growth showing the impact of surface aeration in the shake flask. dCO_2_, dissolved CO_2_; DO, dissolved oxygen; OD, optical density; SF, shake flask [Color figure can be viewed at wileyonlinelibrary.com]

Figure 2 shows the dCO_2_, DO, and optical density (OD) profiles for the 24‐hr *E. coli* culture grown in shake flasks with rubber septum caps. The shake flasks have 0.2 µm sterile air filters acting as exhaust mimicking the standard bioreactor setup. It was observed that with and without intensified surface aeration, there was a significant difference in dCO_2_ and DO concentration. The dCO_2_ was found to be significantly less (>5 times lower) in the shake flasks with air overlay (dCO_2_ < 20%) compared to the shake flask in which there was no overlay. The dCO_2_ was so high in the no overlay condition that the sensor was got saturated and the concentration reached beyond the calibration range. The oxygen‐limitation present in the shake flask with no overlay disappeared in the shake flask with air overlay due to the increased oxygen availability. The faster CO_2_ removal and greater O_2_ availability greatly improved the performance of the shake flask in terms of biomass growth and product concentration. The flask with intensified surface aeration was found to have 36% more biomass growth (8.3 OD) compared to the shake flask without intensified surface aeration, which reached only up to 6.1 OD. The wet cell weight (WCW) was increased by 43% with intensified surface aeration (Table 1). The recombinant protein production was analyzed and found that with air overlay the protein yield was increased by more than five times compared to the case in which there was no overlay kept (Table 1). These results show that surface aeration intensification plays a critical role in O_2_ supply and CO_2_ clearance in shake flasks with rubber septum caps, resulting in improved biomass growth and protein production.

**Table 1:** Recombinant protein production in the shake flask culture with rubber septum caps

Surface aeration	Protein yield (µg)	WCW (g)	Normalized protein yield (%)
No overlay	194.8	0.7	0.028
Air overlay	1587.6	1.0	0.159

Abbreviation: WCW, wet cell weight.

**Figure 3:**
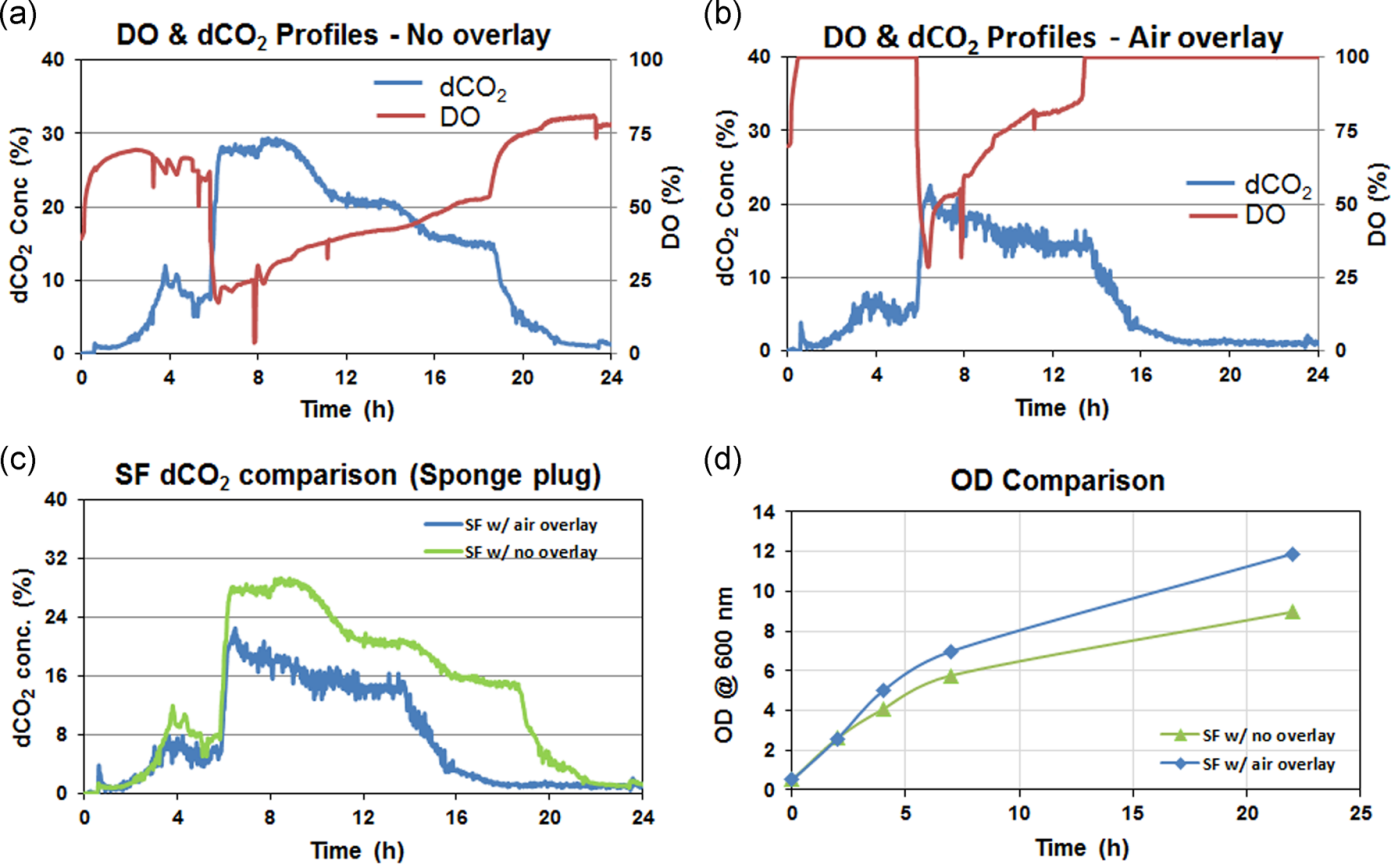
Process parameters monitored in *Escherichia coli* shake flask fermentation with sponge cap. (a) DO and dCO_2_ profiles with no overlay. (b) DO and dCO_2_ profiles with air overlay. (c) Comparison of dCO_2_ profiles under different overlay conditions showing the significance of surface aeration. (d) Comparison of biomass growth showing the impact of surface aeration in the shake flask. dCO_2_, dissolved CO_2_; DO, dissolved oxygen; OD, optical density; SF, shake flask [Color figure can be viewed at wileyonlinelibrary.com]

We further tested the sponge caps as they are more commonly used. Figure 3 shows the dCO_2_, DO and OD profiles for the 24‐hr *E. coli* cultures grown in shake flasks with sponge caps. It was observed that with and without air overlay, there was also a big difference in dCO_2_ concentrations as depicted in Figure 3c. The dCO_2_ was found to be almost 50% less in the shake flasks in which overlay was kept with air compared to the shake flask in which there was no overlay. The sponge cap provided enough oxygen in both cases, evidenced by the absence of oxygen limitation. In contrast, when we used rubber septum with no overlay condition, the DO was reached to zero from 6 to 12 hr (Figure 2). With sponge caps, the flask with lower dCO_2_ concentration found to have 33% more biomass growth (~12 OD) compared to the shake flask having a higher dCO_2_ concentration (~9 OD). The WCW in both conditions with the sponge caps was found to be similar.

We further analyzed the recombinant protein production and found that with the air in overlay the protein yield was increased by 57% compared to the case in which there was no overlay kept (Table 2). This result shows the significance of the dCO_2_ on the culture growth as well as on protein production despite the presence of sufficient oxygen. Surface aeration plays a critical role in CO_2_ clearance from the headspace thereby enhancing the diffusion of CO_2_ out of the broth. The resulting lower concentration of dCO_2_ leads to improved biomass growth and protein production.

**Table 2:** Recombinant protein production in the shake flask culture with sponge caps

Surface aeration	Protein yield (µg)	WCW (g)	Normalized protein yield (%)
No overlay	1357	0.8	0.170
Air overlay	2137	0.8	0.267

Abbreviation: WCW, wet cell weight.

### 3.2 Impact of overlay on fermentations conducted in mini‐bioreactors

**Figure 4:**
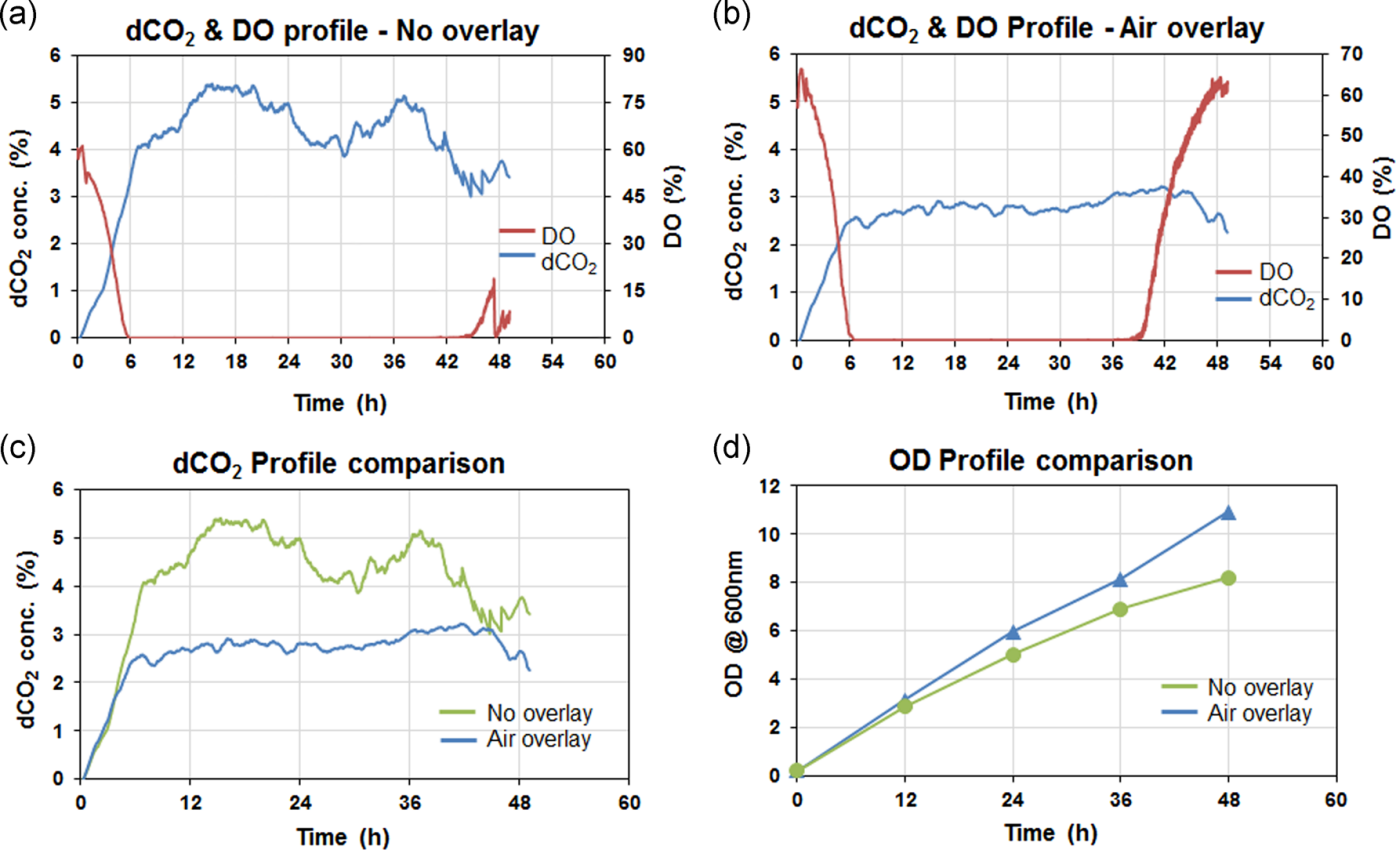
Process parameters monitored in recombinant *Yarrowia lipolytica Po1g Leu* yeast fermentation in mini‐bioreactor (a) DO and dCO_2_ profiles with no overlay (b) DO and dCO_2_ profiles with air overlay. (c) Comparison of dCO_2_ profiles under different overlay conditions showing the significance of surface aeration. (d) Comparison of biomass growth profiles showing the impact of surface aeration in the mini‐bioreactor. dCO_2_, dissolved CO_2_; DO, dissolved oxygen; OD, optical density [Color figure can be viewed at wileyonlinelibrary.com]

One of the most important factors in bioreactor operations is mass transfer, which includes both oxygen supply and dCO_2_ stripping. In the scale‐up of industrial biomanufacturing, dCO_2_ buildup is one of the most serious issues because of its significant impact on the culture condition as well as on the product production. However, excessive stripping of dCO_2_ is also detrimental to cell growth, which suggests that there is likely an optimal level of dCO_2_ for cell culture (Mostafa & Gu, 2003). The organism used for production and the product of interest will certainly determine this optimal dCO_2_ value. Therefore, it is critical to monitor and control dCO_2_ levels. We assessed the impact of keeping the air in the headspace (overlay) of the mini‐bioreactor (50 ml working volume) for *Y. lipolytica* culture grown for 48 hr in batch mode at 30°C (Figure 4). It was observed that when air was sparged in the headspace of the mini‐bioreactor, dCO_2_ concentration was lowered in the culture broth indicating better stripping of dCO_2_ from the vessel. The dCO_2_ concentration with intensified surface aeration was almost reduced to half (2–3%) compared to the no overlay condition. The oxygen‐limitation condition lasted about 6 hours shorter for the culture with air overlay. The lowering in the dCO_2_ levels together with improved oxygen limitation resulted in higher growth and the OD reached above 10 compared to the only 8 OD in the case where no overlay. This indicates that the culture conditions are dependent on the interplay between DO and dCO_2_, which is significantly affected by intensifying air in the overlay of a vessel.

## 4 DISCUSSION

O_2_ supply and CO_2_ stripping are two important factors in determining the success of biomanufacturing scale‐up. They are two of the parameters that are challenging to replicate in the same way at different scales of manufacturing (Sieblist et al., 2011; Xing, Kenty, Li, & Lee, 2009). One can replicate constant glucose or any other metabolite concentration‐based strategy or other scale‐up strategies, but the concentration maintenance of dissolved gases across different scales is challenging due to the complexity of biological systems and the dynamic and hydrodynamic involved in the process (Chopda, Rathore, & Gomes, 2015; Gomes, Chopda, & Rathore, 2018; Persad, Chopda, Rathore, & Gomes, 2013). Researchers have evaluated different strategies to maintain DO supply and low dCO_2_ level such as (1) sparge(a) rate, (2) agitator speed, (3) impeller position, and (4) aeration rate at the headspace of bioreactor. All these methods are tested in standard bioreactor. Some of these like agitator speed and impeller position will not apply to the shake flask and may not be ideal choice to vary for shear sensitive cultures. However, we aim to have a general method which can be applied at all scales from shake flask to manufacturing bioreactor. We have tried sparging the culture in the shake flask, however, it foams out of the flask. Henceforth, we decided to evaluate the impact of sparging in headspace of shake flask, which to the best of our knowledge very few case studies are available on this concept (Takahashi & Aoyagi, 2018c). Further, we have monitored the impact of sparging in the headspace through our novel rate‐based CO_2_ sensor, which measures dCO_2_ concentration in the culture broth.

Surface aeration through air overlay is common in cell culture at large scale to minimize the adverse effect of CO_2_. However, in the small‐scale systems, such as in shake flask and mini‐bioreactor, it is generally assumed that surface aeration through headspace is sufficient for both oxygen supply and CO_2_ stripping. But our results in this study showed that it is not true. Instead, sparging air in the headspace of shake flask and mini‐bioreactor led to improved performance in terms of increased biomass growth and protein production. We also expect that this might be one of the reasons why most of the shake flask experiments are not reproducible at bioreactor level or why scale‐up and technology transfer activity always has to consider a standard benchtop bioreactor. One interesting article (Matsunaga et al., 2009) suggests that it is not always valid to adjust the culture conditions based on only the constant *k*_La_, which is the conventional approach. The evidence observed in CHO cultures shows that the dissolved gases concentration also holds the key to a successful scale‐up (Matsunaga et al., 2009). As a result, it is important to consider surface aeration as a manipulative factor.

A similar impact of headspace aeration on dCO_2_ concentration in the bioreactor has been reported (Mitchell‐Logean & Murhammer, 1997; Mostafa & Gu, 2003). By sparging the air in the headspace, they were able to reduce dCO_2_ concentration from 24 to 6 mM, which resulted in increased cell density for insect cell culture (Mitchell‐Logean & Murhammer, 1997).

A study conducted by McIntyre and McNeil (1997) concluded that culture is more vulnerable to CO_2_ inhibition in the lag phase. In addition, during the inoculation step, the culture can experience many stresses due to the significant differences in the environment of a shake flask and in the bioreactor conditions. Monitoring and regulating dCO_2_ levels will allow us to control the culture environment to prevent any severe shock to the growing cells. Surface aeration intensification significantly enables us to manipulate this gas distribution. We believe that the shake flask and mini‐bioreactor case studies presented in this paper using our proposed aeration modification will be an important factor to be considered in process development and scale‐up activities.

## 5 CONCLUSIONS

Our investigation proved that the surface aeration plays a critical role in shake flask process development. With controlled surface aeration in the shake flask, we were able to not only improve biomass growth but also reach higher protein yield. In addition, this study confirms and demonstrates the application of our novel noninvasive rate‐based in‐situ dCO_2_ monitoring sensor in shake flask and in mini‐bioreactor conditions.
